# Theoretical framework of the neuro and psychomotor therapist for developmental age: an Italian perspective for paediatric rehabilitation of neurodevelopmental disabilities

**DOI:** 10.3389/fmed.2026.1798306

**Published:** 2026-05-05

**Authors:** Giulia Purpura, Andrea Bordin, Alessandra Colucci, Laura D'Arrigo, Andrea Serena Spanevello, Stefania Petri, Fiorenza Broggi, Giulio Santiani, Giorgia Coratti, ANUPI Research Group, Rita Abrunzo, Alice Bernabei, Sara Franchi, Pietro Ilio Gatti

**Affiliations:** 1 School of Medicine and Surgery, University of Milano Bicocca, Monza, Italy; 2 IRCCS Fondazione Don Carlo Gnocchi, Milano, Italy; 3 ANUPI Research Working Group, Associazione Tecnico Scientifica ANUPI TNPEE Ente Terzo Settore, Milano, Italy; 4 Università degli Studi di Pavia, Pavia, Italy; 5 ASL Bari, Operative Unit for Child and Adolescent Neuropsychiatry, Bari, Italy; 6 Presidio Riabilitativo Villa Santa Maria, Vicenza, Italy; 7 Italian Institute of Technology, U-VIP Unit for Visually Impaired People, Genoa, Italy; 8 Department of Informatics, Bioengineering, Robotics and Systems Engineering, University of Genoa, Genoa, Italy; 9 Presidenza, Associazione Tecnico Scientifica ANUPI TNPEE Ente Terzo Settore, Milano, Italy; 10 Pediatric Neurology Unit, Catholic University of Sacred Heart, Rome, Italy; 11 Centro Clinico Nemo, U.O.C. Neuropsichiatria Infantile, Fondazione Policlinico Universitario Agostino Gemelli IRCCS, Rome, Italy

**Keywords:** children, neuro and psychomotor therapist for developmental age, neurodevelopmental disabilities, neurodevelopmental disorders, rehabilitation

## Abstract

**Purpose:**

The rehabilitation of neurodevelopmental disabilities is a complex process aimed at enhancing children’s functional abilities, minimizing the impact of disabilities on family dynamics, and promoting autonomy in daily life. Although scientific literature provides general guidelines, there remains a need for evidence-based therapeutic approaches and for professionals with specialized training in paediatric rehabilitation capable of implementing such interventions effectively.

**Method:**

This paper outlines the theoretical framework underlying the role of the Neuro and Psychomotor Therapist for Developmental Age (TNPEE), an Italian health professional specifically trained to design and deliver evidence-based interventions for individuals aged 0–18 years with neuropsychiatric disorders. The TNPEE operates within a bio-psycho-social perspective, integrating clinical reasoning with developmental neuroscience.

**Results:**

The TNPEE’s professional identity is grounded not merely in methodology but in scientific principles, emerging from the need to address developmental, neurological, and psychological dysfunctions through an integrated, movement-centred approach. This expertise enables the translation of scientific evidence into individualized rehabilitation programs, adapting therapeutic strategies according to each child’s developmental profile, environmental context, and family needs.

**Conclusion:**

TNPEEs are developmental specialists positioned to bridge science and clinical practice. They may play a pivotal role in fostering children’s developmental potential and promoting inclusive, evidence-based care within paediatric rehabilitation.

## 1 Introduction

Neurodevelopmental disabilities (NDDs) encompass a broad range of childhood-onset conditions, often very different among them, that impact neurodevelopment in several domains such as sensory-motor skills, communication, cognitive functioning, and social interaction. Causes may be genetic, lesional, or environmental, often congenital or, less frequently, acquired during the developmental age (1, 2). These conditions include, but are not limited to, autism spectrum disorder, language disorders, cerebral palsy, intellectual disabilities, attention-deficit/hyperactivity disorder, neuromuscular disorders, developmental coordination disorder, sensory impairments, multiple disabilities, and others. Their common features include not only the onset during developmental age, but also the lasting impact of this type of disability on patients and their families throughout life, shaping their growth, adolescence, and transition into adulthood (3–5). From a neurological perspective, these conditions are characterized by alterations in the development and functioning of central and/or peripheral nervous system networks, affecting processes such as sensorimotor integration, motor control, executive functioning, and socio-emotional regulation. Therefore, rehabilitation approaches must be grounded not only in developmental principles but also in an understanding of the underlying neurobiological and neurofunctional mechanisms across the entire sensorimotor system.

For these reasons, scientific literature supports the view that effective and early rehabilitative interventions are a crucial focus in both clinical and research settings for these disorders, although maintaining a life-span approach remains key to focusing rehabilitation on the special needs of individuals at different stages of life (2, 6–9). Paediatric rehabilitation for NDDs aims to enhance children’s functional skills, reduce the impact of disabilities, and promote independence in daily activities. Thus, interventions must be multidisciplinary, multidimensional, tailored to the patient and family-centred, by a team with experts from different disciplines, such as physical therapy, speech therapy, occupational therapy, and psychological support (2, 10).

But *children are not small adults:* they have specific needs and challenges that require a developmental approach, one that takes into account the complexities of chronic disorders in both rehabilitation and education. From this perspective, interventions should not focus solely on modifying body structures and functions to achieve “normal skills” in different developmental domains. Instead, they must aim to support overall child development and foster achievement in whatever form it may take (11, 12). This complex perspective is based on the profound knowledge of the rehabilitation professionals about typical and atypical neuropsychomotor development and its neurobiological underpinnings, but also on the knowledge of the International Classification of Functioning, Disability and Health - Children and Youth (ICF-CY) framework and its application in the structuring of interventions for children with NDD. This bio-psycho-social model provides both a multi-perspective approach to disability as an interactive and evolutionary process and practical fundamentals for practitioners, based on the view that a child’s functioning depends on continuous interactions with the family or other caregivers in a social environment (13). On the other hand, Rosenbaum and Gorter (14) suggested the need to formulate new ways of thinking about children’s needs, as well as child development, and the socio-ecological forces in the lives of children with chronic conditions and their families, integrating the so called “*F-Words*” in child neurodisability – function, family, fitness, fun, friends and future – to traditional ICF-CY approach, to benefit children, parents, families and professionals.

In this context, the scientific tradition of Italian “*Child and Adolescent Neuropsychiatry*,” founded by Prof. Bollea in the 1960s (15), has represented fertile ground for this approach to paediatric rehabilitation. In fact, to address the points explained above, the figure of “Neuro and Psychomotor Therapist for Developmental Age” (in Italian, Terapista della Neuro e Psicomotricità dell’Età Evolutiva – TNPEE) was instituted.

The TNPEE is a healthcare professional with a specific academic bachelor’s degree who develops and implements interventions to establish prevention programs and assesses and rehabilitates individuals aged 0 to 18 with disorders related to child neuropsychiatry (16). Crucially, this profession was born on the principle of psychomotricity, which emphasizes the fundamental importance of the body, physical contact, and relational aspects in child development and intervention.

Within the international context of paediatric rehabilitation, TNPEE can be positioned alongside paediatric physiotherapy, occupational therapy, and speech and language therapy, while maintaining a distinctive developmental and relational focus.

The psychomotor approach underlying the TNPEE work recognizes that motor, cognitive, and emotional development are intrinsically interconnected, viewing the child as a unified being in which movement, sensations, and psychological processes cannot be separated (17). This holistic perspective shapes TNPEE practice through several key principles: the therapeutic use of the body as both medium and message in intervention; the centrality of the therapeutic relationship established through physical presence and contact; the understanding that motor experiences provide the foundation for cognitive and emotional development; and the recognition that play and movement serve as natural vehicles for learning and healing in children. Consequently, TNPEE interventions prioritize experiential and embodied approaches over purely cognitive or mechanistic treatments, emphasizing the child’s active participation in their developmental process through meaningful motor and relational experiences. This is in line with scientific literature that supports the view that intervention should act on perceptual-motor behaviours not only as a means to improve function and participation in the present moment but also as vehicles to facilitate future development across domains broadly and to advance readiness to learn in school (18).

This paper explores the theoretical framework and clinical implications of the TNPEE model, situating it within the broader international landscape of paediatric rehabilitation.

## 2 Neuroscientific theoretical framework of the TNPEE

### 2.1 Origins of the TNPEE as a healthcare rehabilitation professional

The current healthcare profession of the TNPEE originates in Italy from the evolution of the “Re-education Specialist of Psychomotricity,” which was born in the mid-20th century as a technical-auxiliary professional to support child neuropsychiatrists in the education of disabled children. This figure was inspired by the French psychomotricity: in particular, Dupré is rightly recognized as the founder of psychomotricity, due to his studies of children with motor coordination impairments and intellectual disability resulting from developmental dysfunction, in the absence of neurological lesions (19). Subsequently, this approach was enriched by further scientific contributions, particularly thanks to authors such as Wallon (20), Piaget (21), Heuyer (22), and others, who defined the key role of the body as a relational instrument and underlined the close correlation between motor skills, intelligence, and affectivity. The pinnacle of this approach was reached in the 1950s with Ajuriaguerra (23), who integrated neurological and psychological knowledge and valorised the previous studies on psychomotricity to understand and rehabilitate some developmental disorders from a psychomotor evolutionary perspective.

**Figure 1:**
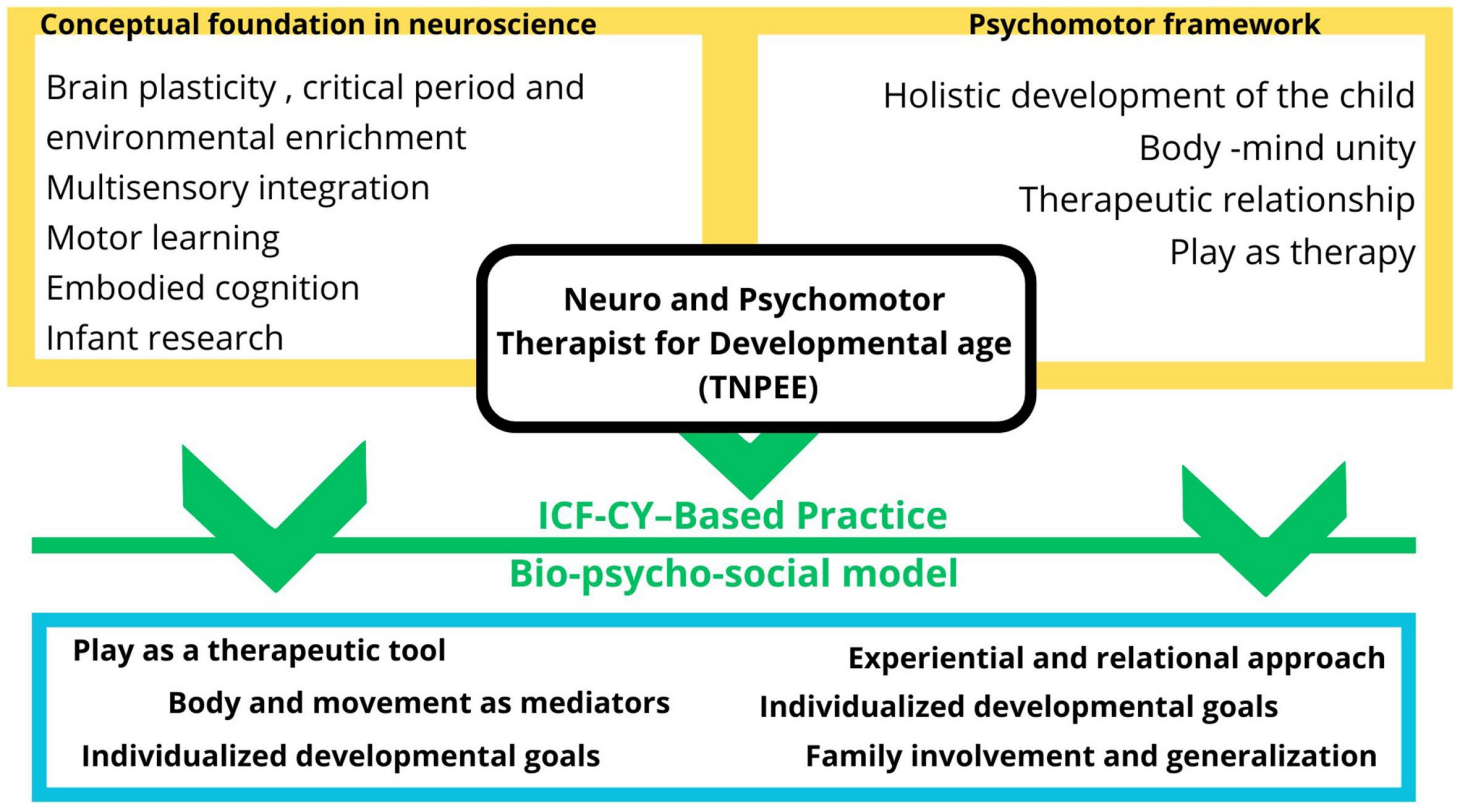
Overview of the theoretical framework for the clinical practice of the TNPEE.

Progressively, from the 1970s, “training schools” for Neuro and Psychomotor technicians were created in several regions of Italy: in this context therapeutic skills from the classical psychomotor approach were integrated with new neurorehabilitation models used by physiotherapists and occupational therapists worldwide for children with cerebral palsy and other developmental disorders, including Bobath’s concept (24, 25), Kabat’s technique (26), Doman-Delacato method (27) and Sensory Integration Therapy of Jean Ayres (28, 29). Gradually, between the 1980s and 1990s, training schools evolved into university-level diplomas, marking the formal recognition of the discipline and aligning the specific education of these professionals with evidence-based medicine. By 2001, university diplomas were further upgraded to bachelor’s degree programs for TNPEE. Thus, the profession evolved to address developmental disorders through an integrated rehabilitation approach (16). To carry out this aim, this discipline has been enriched and continues to be enriched by numerous modern neuroscientific contributions (see Figure 1).

The main theoretical principles underlying TNPEE practice, along with their supporting evidence, are summarized in Table 1 and will be discussed in the following paragraphs.

**Table 1:** Main evidence supporting the theoretical principles underlying TNPEE practice.

Theoretical principle	Key concepts	Clinical implications for TNPEE	Key references
Neuroplasticity and critical periods	Experience-dependent plasticity; sensitive periods; environmental enrichment	Importance of early, intensive, and individualized intervention to support adaptive neurofunctional and neuromotor development, with active involvement of caregivers and environment	(6, 7, 31–33, 40)
Multisensory integration	Integration of sensory inputs (visual, tactile, auditory, proprioceptive, vestibular)	Use of multisensory stimulation embedded in meaningful and ecological activities to support sensorimotor integration and neuromotor function	(27, 28, 42, 43, 46, 51, 58)
Motor learning	Task-specific practice; active engagement; feedback; variability	Emphasis on active participation, task-specific practice, and play-based activities to promote neuromotor control, coordination, and functional movement	(17, 72–74, 77, 78)
Embodied cognition	Interaction between body and cognition; sensorimotor grounding of knowledge	Integration of movement and cognition in therapy, supporting neuromotor and cognitive development through embodied and sensorimotor experiences	(69, 70, 85, 87, 94, 95, 113)
Infant research and intersubjectivity	Early caregiver–infant interaction; co-regulation; attachment	Centrality of therapeutic relationship and caregiver involvement in supporting early regulation processes and sensorimotor development	(96–98, 102, 110)
Play-based development	Intrinsic motivation; symbolic play; social interaction	Use of play as both assessment and intervention tool to promote neuromotor, cognitive, and socio-emotional development within individualized and motivating contexts	(114, 116, 118, 121, 124)

While these neuroscientific frameworks are well-supported in the literature, their translation into specific intervention strategies involves clinical inferences that require independent empirical validation.

### 2.2 Brain plasticity, critical periods, and environmental enrichment

*Neuroplasticity*—the capacity of the central nervous system to reorganize its structure and function and modulate functional connectivity in response to internal and external stimuli—has long been framed as a foundation for learning, development, and recovery (30, 31). However, emerging evidence suggests a more nuanced view: plasticity is not inherently beneficial, and its effects depend critically on timing, context, and the balance of regulation (32–34). While it allows the potential of rehabilitation to be realized, thereby promoting adaptive processes such as learning, it can also underlie vulnerabilities to developmental derailment and, in some cases, to maladaptive or pathological reorganization.

During early life, developmental plasticity sculpts the architecture of neural networks, setting the stage for sensory, motor, and cognitive competencies (35). Critical and sensitive periods represent key windows of opportunity: times when experience can exert lasting effects on brain organization, but also when the brain is most susceptible to adverse influences such as hypoxia, stress, or deprivation (36, 37). Understanding these windows is crucial for designing effective interventions.

Although plasticity persists beyond childhood, providing a substrate for compensatory mechanisms and functional reorganization throughout the lifespan, several studies suggest that it reaches its maximum potential during the early stages of life (31). This aspect indicates that neurorehabilitation may be most effective when interventions coincide with sensitive periods of development (32). In this context, environmental influences are fundamental, and they interact with genetic and neurobiological factors to shape developmental trajectories. Early stimulation programs have demonstrated long-term benefits, particularly involving caregiver engagement (38, 39). Enriched environments, adequate nutrition, and responsive caregiving promote neurogenesis, synaptic efficiency, and resilience—highlighting the potential of non-pharmacological neuroprotective strategies (35, 40–42). In particular, environmental enrichment, through augmented but not invasive sensory-motor, social, and cognitive opportunities of stimulation, profoundly affects the central nervous system at the functional, anatomical, and molecular levels (40).

Thus, plasticity is a valuable brain property, and its expression enables it to be dynamic, modifiable, and shaped by experience and environmental interventions across multiple periods of life. Harnessing neuroplasticity requires more than just promoting change—it demands knowing *when, how, and under what* conditions change leads to meaningful, functional outcomes.

### 2.3 Multisensory integration

Multisensory integration refers to the ability of the central nervous system to combine inputs from different sensory modalities, integrating afferent feedback to support perception and action (43).

This brain capacity underpins adaptive behaviour and socio-cognitive functioning, and its typical development depends on several cortical and subcortical structures as well as innate mechanisms and post-natal experiences (44). These processes rely not only on central integration mechanisms but also on the integrity and modulation of peripheral sensory inputs, including proprioceptive, tactile, and vestibular signals, which play a fundamental role in shaping motor responses and adaptive behaviour. Furthermore, early sensory experiences, particularly through touch and smell, appear to play a key role in parent-infant bonding and the development of co-regulation from the earliest stages of life (45, 46). The application of *multisensory integration* principles in neurological and neuropsychological rehabilitation is closely related to both the fact that multisensory processes facilitate a coherent representation of the environment and its elements, thereby facilitating self-regulation and adaptation to it, and to evidence demonstrating that multisensory experience produces functional brain reorganization through non-invasive rehabilitation strategies (47). The first contributions on the role of multisensory integration in this context come from the pioneering works of Maria Montessori and, subsequently, Ida Terzi. Montessori’s early 20th-century “*scientific pedagogy*” placed sensory experience at the core of cognitive development, arguing that perception is active, selective, and foundational to intellectual growth (48–50). Subsequently, Ida Terzi developed a sensory-motor method for rehabilitating children with visual and motor impairments, based on bodily experiences (51). These approaches anticipated contemporary rehabilitation models by highlighting the integration of sensory inputs and motor experience in shaping development.

More recently, in paediatric rehabilitation, growing evidence indicates that multisensory integration can significantly improve neurodevelopmental outcomes in children with neurological disorders, sensory impairments, or perceptual-motor dysfunctions (44, 52–57). In this context, neuroscientific data also supported the efficacy and usefulness of this approach on brain reorganization (47). For example, randomized controlled trials in preterm infants have demonstrated that multisensory stimulation programs promote healthy development, enhancing feeding efficiency, psychomotor development, and visual function compared to standard care (58). Similarly, the use of sensory-enriched environments, which combine gentle tactile, visual, and auditory stimuli, has shown improvements in motor skills, attention, nonverbal communication, behavioural engagement, and self-regulation in children with severe cerebral palsy, autism spectrum disorders, or other developmental disabilities (59–62). Also, when administered early in development, interventions that couple auditory and motor feedback have been found to improve spatial orientation and perceptual-motor integration in children with visual disorders. Such sensorimotor training helps compensate for visual deprivation by creating robust audio-motor associations that facilitate spatial cognition (63, 64). Moreover, it has been shown that the positive effect of the application of high-tech audiovisual systems, audio-tactile wearable devices, or virtual-reality-based interventions- providing real-time multisensory cues- in educational and therapeutic settings (65–68).

### 2.4 Motor learning

Spontaneous activity is the fundamental expression of the central nervous system (69, 70). Among these, motor activity plays a crucial role in neurodevelopment, as it both requires and promotes the maturation of basic psychological functions essential for environmental adaptation (71). In this context, Von Hofsten (72, 73) emphasizes that action, considered as goal-directed voluntary movement with intent, is a central component of cognitive development, including aspects such as social understanding and environmental exploration. However, *action and exploration are intrinsically reciprocal processes* (71). While perceptual information defines the constraints and affordances of the current environment to guide the action, exploratory movements actively provide the sensory input needed by perceptual systems to function effectively (74).

These complex behavioural processes contribute to a child’s intrinsic predisposition to learn, driven by an innate explanatory tendency to understand their experiences and progressively develop mastery over their environment (75). Based on these points, learning is a spontaneous process, and neuroplasticity underlies its manifestation in the typically developing brain and during re-learning through rehabilitation in the damaged brain (76). In this context, motor learning is the acquisition and modification of learned movement patterns over time and involves cognitive and perceptual processes, as well as the continuous refinement of motor output through afferent feedback (77). From a rehabilitation perspective, these processes are central to neuromotor rehabilitation, as they directly target the organization and refinement of motor control, coordination, and functional movement patterns.

According to the application of motor learning’ concept to rehabilitation, individuals acquire, refine, or re-acquire motor skills through repeated practice and active experience, for first in a controlled space under the guide (verbal, visual, tactile, etc) of the therapist, and successively through a transfer of competence in ecological setting, under the guide of caregivers or through the self-monitoring by the patient own (78). This process relies on active, goal-directed practice, supported by feedback, to promote experience-dependent plasticity (76, 79–84). In the context of motor learning approaches applied to children with NDD, therapists exploit the intrinsic human predisposition for voluntary movement to support the acquisition of novel motor skills in atypical conditions. Therapeutic interventions are systematically adapted to the natural course of the disorder and the child’s specific clinical characteristics. Significantly, motor learning differs from simple performance improvement in that it emphasizes long-term retention and generalization of the skill, not just short-term success. In paediatric rehabilitation, motor learning principles may be adapted to developmental stages and learning styles, since children learn best through play-based, exploratory, and socially engaging tasks that promote motivation and attention.

### 2.5 Embodied cognition

The *Embodied Cognition Theory* emerges from the conceptualization of a profound interdependence between body and mind, whereby both function synergistically to construct human experience and enable cognitive processes (85). This theoretical framework represents a paradigmatic shift from conceiving the body as a mere vessel for the brain to understanding it as an integral and active contributor to cognition (86). Accordingly, from a neuropsychological perspective, the body is no longer conceived as a mere executive apparatus, but as an active medium through which sensorimotor experience shapes perception, cognition, and meaning across development. Consistent with this view, a recent theoretical contribution in this area emphasizes that early bodily exploration supports not only action, but also the emergence of conceptual and symbolic development (87). Within the embodied cognition literature, it is posited that both the motor activities individuals enact and those observed in their surrounding environment exert formative influence on mental representations and cognitive functioning through the mirror neuron system (88). The body is thus seen as instrumental in selecting, structuring, and interpreting sensory inputs that give rise to perceptual and conceptual experiences (89).

This framework aligns with evidence suggesting that motor and mental activities reciprocally influence one another through dynamic sensorimotor loops linking perception, action, and cognition throughout neurodevelopment. Indeed, motor and cognitive development cannot be considered separate processes, since, from intrauterine life onward, the relationships between movement and perception play a key role in the individual experience (90–92). For example, Babik and collaborators (93) highlighted that the amount of self- and object-exploration behaviour performed is significantly related to children’s motor, language, and cognitive development throughout the first two years of life. Similarly, whole-body movements in numeracy have been demonstrated to increase engagement and understanding among elementary students (94).

In preterm infants at risk of developmental disorders, early interventions that promote the child’s initiation of exploratory and investigative movement importantly serve the early learner to build problem-solving skills, while advancement of motor skills without including strategies that build problem-solving, self-initiated movement, and object exploration may have adverse effects on cognitive advancement (95).

### 2.6 Infant research

The term *Infant Research* refers to a field of study that emerged in the late 1960s, characterized by the adoption of increasingly refined observational and experimental methodologies—most notably microanalytic procedures—to investigate early developmental processes (96). This body of research marked a significant paradigm shift: from viewing the infant as a self-contained organism endowed with individual capacities, to understanding the infant as a relational being, shaped by—and actively shaping—early interactions with caregivers. At the heart of this approach lies the study of the infant–caregiver dyad, particularly the infant–mother relationship, as a primary context in which affective communication, mutual regulation, and the foundational sense of self take shape. Detailed, moment-by-moment analyses of nonverbal exchanges have enabled researchers to uncover how early interactions contribute to the development of attachment, intersubjectivity, and the psychological architecture of the self (97). Seminal contributions to this field have come from scientists such as Daniel Stern (98), Colwyn Trevarthen (99), Edward Tronick, Beatrice Beebe and Frank Lachmann (97), and Allan Schore (100), and others. However, Donald W. Winnicott (101) was among the first to emphasize the vital role of early relationships in fostering the child’s individuality, highlighting the importance of a stable and “holding” environment for healthy psychological development. Several core principles have emerged from this relational and developmental framework: (i) the infant is an active subject from the earliest stages of life, capable of perceiving, responding to, and initiating interactions; (ii) early caregiver–infant interactions are fundamental to psychological and emotional development; (iii) the sense of self emerges through distinct relational phases and experiences; (iv) implicit memory stores early relational experiences, creating internal “representations” that influence future relationships; (v) the caregiver’s capacity to modulate and respond appropriately to the infant’s emotional states is crucial for the development of emotional regulation and secure attachment. These early, dynamic interactions are mediated mainly through nonverbal social and emotional exchanges, described as *intersubjectivity*—the shared understanding and mutual engagement that serve as precursors to verbal communication (102).

Primary intersubjectivity refers to the infant’s innate ability to coordinate gaze, vocalizations, facial expressions, and gestures with those of a caregiver (103). Within this framework, Trevarthen (104) introduced the concept of proto-conversation: spontaneous, synchronous exchanges between infant and caregiver involving eye contact, vocal rhythms, gestures, and bodily movements that express mutual emotional awareness and intention. Around 9 to 12 months of age, secondary intersubjectivity emerges. This phase involves joint attention—the triadic coordination between infant, caregiver, and external objects or events. It includes sharing attention, emotions, and intentions regarding the external world (102).

Daniel Stern (105) emphasized that during playful interactions marked by affect attunement, the expressive movements of both parent and infant become synchronized, forming an embodied dialogue. These exchanges reflect the “rhythms of intention” and are central to the infant’s emerging narrative sense of self. This perspective also underscores how early distress can be mitigated through the caregiver’s sensitive responses, which support the infant’s evolving intersubjective capacities. As Trevarthen (106) noted, children learn language and develop cognitive-emotional skills by engaging in shared activities filled with invention, discovery, and mutual delight.

## 3 The child from the perspective of the TNPEE

When we talk about paediatric rehabilitation, it is essential to remember the intervention’s proper focus: the child and their family. In particular, children are individuals in the making, with their own biological and psychological peculiarities specific to the developmental period they are in. As highlighted in the ICF-CY:

"[…] in children and adolescents, the manifestations of disability and health conditions are different, in their nature, intensity, and impact, from those of adults […]".

In line with this concept, a key point for the TNPEE is the entire theoretical and practical training on the distinctive characteristics of childhood (16).

According to the TNPEE’s approach, different key domains of neurodevelopment- sensory-motor, cognitive, socio-emotional, communicative-linguistic, and behavioural- are deeply interconnected, and their interactions define each child’s individuality (107). For these reasons, they cannot be analysed and educated separately, and understanding typical development is essential for monitoring progress and identifying potential delays or challenges. Although several observational studies and theoretical models have identified shared patterns in the sequence and timing of skill acquisition, typical development is characterized by significant adaptive variability in times and strategies of learning. In fact, human behaviour shows variability that supports flexible adaptation to the environment (70, 108). Accordingly, the term “*habilitation*” is often preferred over “rehabilitation” for childhood-onset disorders, as it more accurately refers to the initial acquisition of abilities that were never developed, rather than the recovery of previously acquired but subsequently lost functions.

In the same way, the centrality of context and environment is crucial for the holistic and ecological care of the child with neurodevelopmental disability. Once again, the biopsychosocial perspective of the ICF framework highlights:

"[…] a person's functioning and disability are conceived as a dynamic interaction between health conditions and contextual factors […]".

The ICF (13) framework recognizes that biological factors are only one part of the equation, and psychosocial factors are implicated in the aetiology and treatment of many physical and mental health conditions (109). Moreover, the relationship between the child and his/her environment is not unidirectional, but reciprocal. As Piaget (110) emphasized, children are not passive recipients of external stimuli but active participants in their development. The world influences the child, just as the child influences the world. This dynamic interaction is the foundation of developmental maturation (111), and direct experience and active engagement with the world are essential for the child’s understanding of others’ actions, intentions, and emotions (112).

This is especially relevant in paediatric rehabilitation, since the child’s development is closely tied to their social environment, including family and caregivers, as well as friends, educators, and teachers. The social and environmental contexts in which a child lives play a vital role in shaping their growth. Movement, play, and social relationships are a child’s world’s most compelling elements because they provide opportunities for pleasure, self-competence, and environmental connection (113). For an intervention to be effective, these ecological aspects must be considered, understood, and actively involved in the rehabilitation process of children with neurodevelopmental disabilities. Furthermore, recent scientific literature found that visual and motor imagery, far from being of a disembodied, make use of the activation of sensory-motor brain regions; in this context, sensory-motor regions are also exploited for “high-cognitive functions,” for example the characterization of the so-called “abstract” concepts, which are the elementary units of reason and linguistic meaning (114).

## 4 Play as a key tool for enrichment and habilitation

Play has long been a central focus in developmental psychology studies because it has been widely demonstrated to shape children’s growth and play a key role in healthy physical, social, emotional, and cognitive development (115). Not by chance, Jean Piaget already theorized a connection between stages of intellectual development and the organization of play, emphasizing the balance between assimilation and accommodation processes (110, 116). Within this framework, play reflects a fundamental process in child development, supporting cognitive, emotional, and social growth. It evolves across developmental stages and provides a natural context for learning and exploration (117).

In fact, its core characteristics include intrinsic motivation, focus on process rather than outcome, freedom from external constraints, and the creation of symbolic realities. Play allows children to transcend the limits of the real world and enter imaginary scenarios in which they rehearse daily-life roles, explore emotions, and experiment with social rules. This dynamic, self-directed, and non-linear nature of play makes it uniquely suited to meet each child’s individual developmental needs, providing learning opportunities that are both spontaneous and deeply personal (118). Moreover, social play stimulates the development of executive functions—including inhibitory control, cognitive flexibility, and working memory—critical for adaptive behaviour. These skills enable children to manage impulses, plan actions, shift attention, and modulate emotional responses, which are crucial for success in daily life, school, and interpersonal relationships (119).

As Rosenbaum (14, 120) sustains in the already cited “Five Words” for child neurodisability, the dimension of *Fun* is crucial in rehabilitation. Fun is embedded in interventions performed by TNPEE to engage children in motivating contexts that foster exploration, learning, and intrinsic motivation. The TNPEE supports the natural learning style by designing highly personalized activities within playful contexts to achieve individualized therapeutic goals. Using play sessions, therapeutic work occurs within what Vygotsky (121) termed the “zone of proximal development”—the range of skills beyond the child’s current abilities but attainable with guidance. In this view, learning is experiential and driven by the child’s spontaneous initiatives and intrinsic interests, which form the foundation for creating opportunities to teach new skills. This way of using play by the TNPEE was inspired by the psychoanalytic play-based therapy of Winnicott (122), the floor-time approach of Greenspan (123), and the UK tradition of non-directive play therapy (124). In TNPEE’s practice, both assessment and therapy are based on observing and participating in spontaneous play, which is the most ecological strategy for enhancing the child’s global competencies and supporting healthy developmental maturation. This is also in line with recent scientific literature that showed interventions involving active play components tend to be more effective in improving cognitive and executive functions (125, 126), motor learning (82, 127), and social-communication skills (128, 129).

Furthermore, in children with or at risk of neurodevelopmental disabilities, atypical play behaviors may be observed as early as the first year of life. These differences are not merely developmental delays but often intrinsic expressions of the disorder (115). These children may exhibit repetitive behaviours, reduced symbolic play, poor motor coordination, or difficulty initiating or sustaining social play (118). Consequently, play becomes impoverished in quality and variety, losing its role as a space for exploration and learning. This can have long-term repercussions for emotional development, relational competence, and the acquisition of independence (130). For these reasons, play may be both a means and an end in the habilitation process. It serves as an assessment tool, providing insights into a child’s strengths and vulnerabilities, and as a therapeutic modality, allowing practitioners to guide the child toward targeted developmental goals safely and engagingly (131). The therapeutic space of the TNPEE becomes a protected and responsive environment where the child’s spontaneous activity is observed, supported, and gradually enriched (16). Free play allows the therapist to assess emerging abilities and relational patterns, while guided or co-constructed play enables more structured intervention, with therapeutic goals—such as postural control, attention shifting, and emotional co-regulation—pursued in a non-intrusive, playful manner. Finally, the TNPEE supports the family system by helping caregivers understand the child’s communicative signals, adapt their responses, and recreate meaningful play interactions at home. This integrative approach reinforces the generalization of skills learned during therapy and fosters a collaborative alliance that benefits the child’s overall development (132).

## 5 Body and movement in neuro and psychomotor therapy

The body and movement are fundamental and irreplaceable tools in the clinical practice of the TNPEE. They represent both the child’s primary mode of expression and the main channel for therapeutic interaction. From a neurophysiological perspective, these processes involve the continuous interaction between central motor planning and peripheral execution systems, including muscle activation, proprioceptive feedback, and sensorimotor loops. This bidirectional exchange between central and peripheral levels is essential for the development of coordinated, adaptive, and meaningful action.

Within this perspective, TNPEE practice can also be framed within neuromotor rehabilitation, integrating motor function with relational and developmental processes (133, 134). It is through bodily experience that the child begins to make sense of the world, and this embodied engagement offers a motivating and developmentally appropriate point of entry for therapeutic work. Interventions are grounded in movement and relational dynamics, using play and interactive methods to enhance both motor competencies and the child’s ability to engage meaningfully with their environment (17, 135).

One core strategy for establishing connection through movement is imitation, which serves as a nonverbal signal of interest, attunement, and recognition (136, 137). By imitating a child’s actions, the therapist conveys engagement not only with what the child is doing, but with who the child is as a person (138). In early development, before the emergence of verbal language, the body is the primary vehicle of expression (135). Motor activity is not merely a set of physical actions but also a carrier of emotion, experience, and identity. From a psychomotor perspective, movement is never neutral: it reflects tone, relational quality, and the child’s developmental history, emerging from the integration of central motor planning and peripheral motor output (17). Within this framework, the therapist does not remain a passive observer but engages physically with the child to establish resonance, co-create symbolic play, and support global development (98, 139). Through *bodily play*, neuropsychomotor therapy engages developmental processes across multiple functional domains, helping the child discover effective strategies for relating to the external world and carrying out daily activities. The therapist’s body becomes a therapeutic tool—like space, time, and play materials—forming part of the therapeutic setting. The TNPEE maintains an active, responsive, and empathic presence, grounded in the concept of *bodily and affective resonance* (139). The therapeutic relationship is constructed in the “here and now” through shared gestures, mirroring, and rhythmic interaction (140).

A conscious and attuned use of the body allows the therapist to modulate the relational climate, support the child’s emotional regulation, and foster sensory-motor integration. Shared movement also helps structure time, organize space, and strengthen the child’s perception of the other as a meaningful presence (141). In face-to-face interaction, the space between the child and the therapist is filled with expressive, embodied signals that connect senses and actions, weaving a web of non-verbal communication. The processes of *tonic and emotional co-regulation*—mediated by eye contact, posture, gesture, and rhythm—play a pivotal role in the therapeutic context. From this perspective, movement conveys affective and cognitive meaning, and its harmonious integration becomes a central therapeutic goal. TNPEEs use the body to facilitate the emergence and processing of emotions that are not yet accessible through language. Symbolic movement-based activities—such as jumping, balancing, rough-and-tumble play, or rolling—offer children opportunities to explore and express core emotions (e.g., anger, fear, joy, anxiety) in a shared, protected, and meaningful context (142). In this way, the body also serves as a *symbolic mediator*. In conclusion, the body and movement are not merely areas for assessment or targets of intervention; they constitute the foundation of the therapeutic encounter. Through their embodied presence, TNPEEs create a non-verbal dialogue that is essential for supporting the child’s development in a holistic and respectful way, attuned to their evolving capacities and relational needs (105, 135).

The theoretical principles outlined above reflect a body of knowledge developed across neuroscience, developmental psychology, and rehabilitation sciences, which has historically contributed to the emergence of the TNPEE profession. However, their application to clinical practice is not direct. Therapeutic approaches, including that of the TNPEE, should therefore be understood as interpretative and context-dependent ways of operationalizing these principles, rather than direct translations of scientific evidence.

## 6 Positioning TNPEE in developmental care: clinical applications and future direction

Having outlined the neuroscientific and theoretical foundations of TNPEE practice, we now examine how this professional profile can be situated within the broader landscape of developmental care. In particular, this section explores areas of convergence, specificity, and partial overlap with adjacent professions worldwide and considers how these roles may collaborate across different healthcare systems. This perspective also allows reflection on how specific competencies developed within TNPEE training may inform academic pathways, particularly in relation to developmental, relational, and embodied approaches in paediatric rehabilitation, contributing to international dialogue on professional competencies.

The central point of the TNPEE approach lies in the unique neurobiological characteristics of the developing child, in which growth processes are profoundly sensitive to both enriching and limiting experiences. Developmental domains (motor, cognitive, sensory, emotional, and social) do not evolve in isolation but in constant interaction. A child’s family, cultural, and social environments further shape developmental opportunities and challenges. Finally, the time-sensitive nature of critical and sensitive periods makes evident the importance of timely and appropriate intervention.

These considerations highlight the relevance of expertise in both developmental science and its clinical application, while also prompting reflection on how these competencies are conceptualized, distributed, and operationalized across different professional profiles. Within this context, the TNPEE profession can be understood as a structured and developmentally oriented professional configuration within the broader landscape of paediatric rehabilitation, integrating insights from neuroscience, developmental psychology, and clinical practice. How this configuration compares to other professional models in terms of effectiveness and appropriateness remains an important research question, although the TNPEE already represents a formally established and operational professional role within the Italian healthcare system.

### 6.1 TNPEE within the Italian healthcare system: organisational context and professional role

The role of the TNPEE is closely linked to the organizational and historical structure of the Italian National Health Service (Servizio Sanitario Nazionale, SSN). In Italy, developmental rehabilitation is embedded in a structured care pathway that begins at diagnosis and continues through long-term management, allowing rehabilitation to be adapted over time in response to developmental trajectories and functional changes. Within this system, rehabilitation is organized through different levels of intensity and duration, as outlined in the National Guidelines for Rehabilitation Activities established by the Ministry of Health (143), which define rehabilitation as a structured and integrated socio-health process aimed at developing all the individual’s potential resources and ensuring their social inclusion and quality of life across the lifespan. This framework explicitly encompasses long-term projects for significant disabilities with possible permanent outcomes. The Italian organization of services has historically placed paediatric rehabilitation within child neuropsychiatry, favouring an integrated clinical model in which neurological, developmental, and psychological dimensions are addressed together. This context is essential to understanding the TNPEE role.

As outlined above, the birth of the TNPEE as a formally recognized profession was itself a product of this integrated tradition (see Section 1). It emerged from the convergence between the practice of psychomotricity, which had been developing in Italian educational and therapeutic settings since the 1960s, and the scientific and institutional framework of Italian child neuropsychiatry (NPI). This convergence was promoted by the Associazione Nazionale Unitaria Psicomotricisti Italiani (ANUPI), today Associazione Nazionale Unitaria Terapisti della Neuro e Psicomotricità dell’Età Evolutiva Italiani (ANUPI TNPEE), which recognized the need to integrate psychomotor competencies with the developmental, neurological, and psychopathological knowledge developed within the NPI tradition. The decision to formally anchor the profession to child neuropsychiatry, rather than to a purely psychomotor or educational model, was a defining cultural and institutional choice, and it helps explain the broad scope of intervention that characterizes the TNPEE within the Italian health system.

The TNPEE is formally recognized among the Italian rehabilitative health professions by Ministerial Decree no. 56 of 17 January 1997, alongside professions such as physiotherapy and occupational therapy. The Core Competence of the profession, as defined in the professional literature, covers three interconnected areas: rehabilitative activities targeting impairments in mental, sensory, and neuro-musculoskeletal functions; habilitation activities aimed at fostering the emergence of competencies across learning, communication, mobility, and interpersonal domains; and prevention activities aimed at averting atypical developmental trajectories in at-risk situations and reducing the risk of social exclusion. These three dimensions coexist within a single clinical framework in which intervention is directed not merely at the child but at the entire system—family, environment, and social context—with the goal of supporting an integrated developmental project throughout the whole arc of growth. This approach was also recognized at the academic level, as training to become a TNPEE is a specific bachelor’s degree within the rehabilitation health professions, with a final national licensing exam and a thesis, such as bachelor’s degrees in physiotherapy, speech therapy, occupational therapy, and others.

A recent national survey (144) further documented this breadth of clinical application, demonstrating that TNPEE professionals (to date ~6,000 in the national territory) intervene across a wide range of conditions spanning cerebral palsy, genetic syndromes, neuromuscular diseases, language disorders, and sensory impairments. These data are valuable in delineating the clinical reach of the profession within the Italian healthcare system, whereas questions concerning comparative outcomes across different professional models remain to be addressed through dedicated investigation.

In practice, TNPEEs may work either within multidisciplinary teams or, in some contexts, as the primary rehabilitation provider in the field of child neuropsychiatric disorders. Available Italian sources describe them as part of territorial teams able to form an intervention centred on neurodevelopment, the child–caregiver system, and the generalization of competencies across everyday contexts. At the same time, collaboration with other professionals remains crucial: for example, during specific developmental phases, paediatric physiotherapists may be more directly involved when significant orthopaedic or musculoskeletal impairments are present, whereas occupational therapists may be particularly relevant for assistive devices, environmental adaptations, and activities of daily living. These distinctions between clinicals, while clinically useful, should be understood as tendencies rather than fixed boundaries, highly dependent on each child’s needs, the individual clinician’s expertise, and local service configuration.

This organisation also helps explain why the TNPEE may appear to cover a broader share of developmental rehabilitation responsibilities within the Italian system than in international comparisons. Such breadth may reflect the way competencies are allocated within the SSN, shaped by a historical decision to integrate developmental, neurological, and relational dimensions into a single professional figure rather than distributing them across separate specializations. An example of this decision and of its application is illustrated by the recent Italian RAPIDS survey in Dravet syndrome (145), in which speech therapy and TNPEE were prescribed for almost all children, whereas occupational therapy and physiotherapy were rarely used.

Within the Italian healthcare system, TNPEE represents an established and operational component of paediatric rehabilitation pathways. This professional is included in clinical practice, referenced in national guidelines, and actively involved in multidisciplinary working groups, and in some contexts, depending on the child’s needs, may constitute the primary rehabilitation professional or “case manager” within the child’s care pathway (146–153).

### 6.2 Comparative perspectives on TNPEE within paediatric rehabilitation

Building on this context-specific organisation of competencies within the Italian healthcare system, TNPEE can be examined in relation to adjacent professions in the broader international landscape. Several core elements emphasized in the TNPEE practice (such as play, sensorimotor development, and family involvement) are also well-established in other healthcare professions, such as paediatric occupational therapy and physiotherapy. The distinction lies in how these components are organized and prioritised within each professional framework, not in their exclusive ownership by any single profession.

Contemporary developmental science describes child development as the result of dynamic interactions within and between neural and behavioural systems, rather than as a set of independent functional domains. This perspective informs TNPEE clinical reasoning, insofar as neuromotor function is understood in relation to cognition, affect, and social interaction, and reciprocally influenced by these processes. At the same time, it is important to note that this theoretical orientation, grounded in systems neuroscience and developmental psychology, does not, by itself, constitute evidence for the effectiveness of any specific therapeutic approach or professional model. Other professional frameworks, including but not limited to those of paediatric physiotherapy and occupational therapy, draw on overlapping theoretical foundations while translating them into different clinical priorities and intervention strategies; the relative merits of these different translations are not resolvable on theoretical grounds alone.

From a clinical perspective, a child presenting motor difficulties in the preschool period may be approached by different professionals through different but complementary clinical lenses, each with its own depth and focus. A physiotherapist brings particular expertise in biomechanical analysis, postural control, and neuromotor rehabilitation protocols, areas where a well-developed evidence base exists. An occupational therapist contributes depth in participation-based assessment, assistive technology, environmental adaptation, and activity analysis, drawing on frameworks that are well-established internationally. A TNPEE professional works within a framework in which motor, relational, cognitive, and sensory dimensions are understood as co-determining developmental processes, and in which intervention is organized around the child’s global developmental trajectory rather than around a primary functional domain. All three profiles draw on overlapping evidence-based approaches (e.g., goal-directed training, sensory integration techniques, play-based methods), and the question of which professional is best placed to lead or contribute to a given clinical situation depends on the specific phases in the natural history of the disorder, but also on the team composition and the service context.

An additional distinctive element of TNPEE lies in its training structure, as outlined in a recent paper by Purpura and Coratti (16): the TNPEE education is specifically tailored to developmental age and provides sustained, specific training for interventions in the paediatric populations. This deeply focused training pathway is intended to support the development of integrated competencies across developmental domains, both during theoretical teaching and internship experience. From a training design perspective, this approach may provide useful insights for international discussion on how competencies in paediatric rehabilitation are distributed across professional roles, to ensure a comprehensive and integrated approach to the care of children with disabilities. How this training model compares paediatric specialization pathways in other health professions in terms of measurable clinical outcomes remains an important question for future research.

The relevance of this integrated model has begun to attract attention beyond Italian borders. A comparative analysis of developmental specialists across European countries (154) highlighted significant disparities among European systems in how child disability is managed, both in terms of healthcare organisation and the professional figures responsible for developmental rehabilitation. In several countries, equivalent competencies are distributed across separate roles—other denominations of psychomotor therapists operating primarily within educational or prevention settings, physiotherapists in healthcare, speech therapists in both—without a single figure holding a unified developmental mandate within the healthcare system. This variability underlines the extent to which professional models are context-dependent, shaped by national healthcare architectures and historical traditions, and makes straightforward comparisons between the TNPEE and its European counterparts difficult: the TNPEE profile was constructed in response to the specific structure of the Italian SSN and the NPI tradition, and its transposition to other contexts would require adaptation rather than direct equivalence. At the same time, this variability points to the potential relevance of the TNPEE’s framework as a documented case for international discussion, not as a model that must be exported wholesale, but as an example of how a developmentally integrated clinical profile can be formally embedded within a public health system, alongside other well-established professions, or as an educational framework for healthcare professionals working in paediatric rehabilitation.

A defining characteristic of the TNPEE’s approach lies in its professional identity, which is grounded in a neuroscience-derived framework rather than confined to a single therapeutic method. It relies on an evidence-informed approach that guides the critical, integrated, and targeted use of diverse intervention strategies within the broader perspective of typical and atypical development. This also requires acknowledging that professional boundaries in paediatric rehabilitation are not fixed by training alone. Like physiotherapists and occupational therapists, TNPEE professionals develop specialized competencies through postgraduate training, clinical experience, and continuing education in areas such as neuropsychological assessment, augmentative and alternative communication, early intervention, the knowledge regarding specific diagnostic populations, or particular intervention approaches, that evolves on the basis of science evolution (e.g., DIR/ Floortime, Sense and Mind, ABA, CIMT, Bobath, ESDM, others). It is the responsibility of each clinical professional to select and adapt them to the child’s characteristics and family context, based on evidence-based practice principles. Thus, what the TNPEE model contributes is a professional framework in which developmental integration is the explicit organizing principle from the outset — but this does not preclude, and typically coexists with, deep specialization in particular domains or populations.

In clinical practice, these principles are translated into structured yet flexible intervention processes. Typically, TNPEE interventions begin with a comprehensive assessment that integrates standardized and domain-specific tools with the observation of spontaneous play and relational dynamics, in line with the ICF framework. Based on this assessment, individualized goals are defined across multiple developmental domains. Intervention sessions are then designed using play-based, multisensory, and task-oriented activities that actively engage the child and are progressively adapted in complexity. Particular attention is also given to the generalization of acquired skills through the involvement of caregivers and the adaptation of everyday environments, ensuring ecological validity and continuity of the therapeutic process. As in most clinical settings nowadays, to effectively address the complexity of developmental disabilities, a multidisciplinary clinical practice is typically required also including other rehabilitation professionals (e.g., paediatric physiotherapists for orthopaedic comorbidities or also the psychologist for specific emotional difficulties or for family support) to deliver a unified, person and need-centred intervention. Within this framework, each professional evaluates the system through a distinct clinical lens, targeting specific functional domains while maintaining a shared vision of the child’s complexity.

This view on rehabilitation approaches also reflects the Italian tradition of non-separation between child neurology and child psychiatry (15). However, even within this field, there has been a longstanding discussion whether integration of skills across domains is best achieved through a single comprehensive professional figure or through effective coordination among specialists — a debate that the TNPEE model engages with explicitly, by making developmental integration the organizing principle of the profession from the outset rather than an outcome of coordination.

The TNPEE can be positioned within paediatric rehabilitation alongside other professions as one structured and institutionally grounded configuration of competencies. The differences among the different healthcare profiles should be understood as reflecting distinct ways of organising developmental knowledge and clinical priorities, each with its own strengths, limitations, and evidentiary standing. To give one concrete example: in children and adolescents experiencing pain related to musculoskeletal complications (such as those associated with spasticity, scoliosis, or prolonged immobility) paediatric physiotherapists bring specific competencies in pain assessment and management, therapeutic exercise, and physical modalities that are central to clinical care and that fall outside the primary focus of TNPEE practice. Similarly, in adolescents where the clinical priority is the acquisition of functional autonomy in daily living (including self-care routines, meal preparation, community navigation, or workplace readiness) occupational therapy frameworks offer structured, occupation-based assessment and intervention tools specifically designed for this transition phase, which go beyond what the TNPEE developmental framework addresses as its primary domain. These are not limitations of the TNPEE model but reflect its different clinical orientation, directed primarily toward the developmental processes of childhood and early adolescence. They also underscore why multidisciplinary collaboration is not optional but structurally necessary across the lifespan of children with neuropsychiatric disabilities.

Another important consideration is the broader healthcare landscape in the field of early intervention. In this context, the increasing demand for professionals working with infants and young children highlights the complexity of developmental care in the earliest stages of life, where rapid neurodevelopment, high plasticity, and the interdependence of motor, cognitive, sensory, and relational processes require integrated, timely approaches (155, 156). Particularly in this field, evidence in paediatric rehabilitation is largely associated with specific intervention approaches and developmental mechanisms rather than with individual professional categories (11, 75–77, 157, 158). Consequently, different professions may operate within shared evidence bases, while differing in how these are organised, prioritised, and translated into clinical practice. A more child-centred approach with complex needs has long been advocated by the scientific community, which asks professionals to go beyond domain-specific skills and to embrace the ICF bio-psycho-social approach more fully (11, 158, 159).

Despite its strengths, this framework presents several limitations that should be acknowledged. The TNPEE profession is primarily established within the Italian healthcare system, which may limit its international recognition and comparability with other rehabilitation models. Empirical studies specifically evaluating the effectiveness of TNPEE-led interventions remain limited (130, 132, 160–165). Furthermore, variability in training pathways and clinical implementation may affect the standardization and reproducibility of interventions. As Purpura and Coratti have noted, the absence of a standardized international equivalent complicates professional comparisons and mobility, a challenge that is simultaneously an obstacle to recognition and an opportunity to contribute a distinctive perspective to global rehabilitation science (16).

In conclusion, the TNPEE model represents a distinctive, developmentally focused approach to paediatric rehabilitation. Its conceptual coherence, historical rootedness in the Italian NPI tradition, and breadth of clinical application within the SSN make it a relevant case for analysis within the international landscape. Whether and to what extent this framework translates into measurable clinical outcomes remains an important question for future research. Addressing this question will be essential both to strengthen the empirical basis of the TNPEE practice and to position this clinical contribution within an international evidence base that evolves across professional boundaries.

## Data Availability

The original contributions presented in the study are included in the article/supplementary material, further inquiries can be directed to the corresponding author/s.

## Group member of the ANUPI Research Group

Rita Abrunzo, Alice Bernabei, Sara Franchi, Pietro Ilio Gatti.
